# Correlation Between In Silico Docking/Simulation Results and In Vitro MAGL Inhibition Potency of Selected Triterpenes

**DOI:** 10.3390/cimb47090691

**Published:** 2025-08-27

**Authors:** Willias Masocha, Mohammed A. Khedr

**Affiliations:** 1 Department of Pharmacology and Therapeutics, College of Pharmacy, Kuwait University, Kuwait City 13110, Kuwait; 2 Pharmaceutical Chemistry Department, Faculty of Pharmacy, Helwan University, Cairo 11795, Egypt; mohammed_abdou0@yahoo.com

**Keywords:** MAGL inhibition, endocannabinoids, molecular docking, molecular dynamic simulation, triterpenes, pristimerin, in vitro, screening, correlation, 2-arichdonyl glycerol

## Abstract

Monoacylglycerol lipase (MAGL) degrades the endocannabinoid 2-arachidonyl glycerol. MAGL inhibitors, such as the triterpene pristimerin, alleviate neuropathic pain in animal models. In silico studies were carried out using SwissDock, PyRx-0.8 and CB-Dock2, to check if they correlated with the in vitro MAGL inhibition potency of various triterpenes. In terms of affinity, free energy of binding and docking scores to MAGL, pristimerin (52.75, −9.32, −10.83, and −11.5 kcal/mol) was better than euphol (44.86, −8.49, −9.56, and −10.7 kcal/mol), which in turn was better than β-amyrin (35.17, −7.37, −8.21, and −8.8 kcal/mol). Finally, β-amyrin was better than or equal to α-amyrin (35.10, −7.19, −7.95, and −8.6 kcal/mol). In molecular dynamic simulations (MDSs), pristimerin exhibited the highest stability and reached the steady state after 20 ns with the lowest root mean square fluctuation (RMSF) at the binding site, compared to the triterpenes. The reported half maximal inhibitory concentration (IC_50_) values of recombinant human and rat MAGL inhibition were in the following order: α-amyrin > β-amyrin > euphol > pristimerin. Linear regression analysis showed that the affinity, free energy of binding, and docking scores significantly correlated with the IC_50_ of MAGL inhibition. Amongst the triterpenes studied, pristimerin was the most potent inhibitor of MAGL and also had the highest affinity in the in silico studies. Thus, molecular docking and MDS results correlated with the potency of triterpenes inhibiting MAGL activity in vitro and could be used for screening of triterpenes prior to experimental validation.

## 1. Introduction

Monoacylglycerol lipase (MAGL) is an enzyme that degrades 2-arachidonoylglycerol (2-AG), which is an endocannabinoid [1,2,3]. 2-AG is the most abundant endocannabinoid, an agonist at both cannabinoid type 1 (CB1) and CB2 receptors and is involved in various physiological functions including retrograde signaling in regulation of synaptic transmission and plasticity, inflammation and pain sensation [4,5]. It is produced on-demand and rapidly metabolized by MAGL [4,6]. It has recently been shown that there is a deficiency in 2-AG in the paw skin of mice with paclitaxel-induced mechanical allodynia, which is a model of chemotherapy-induced neuropathic pain [7]. This suggests that inhibition of MAGL could increase the amount of 2-AG and alleviate paclitaxel-induced mechanical allodynia. Indeed, treatment with MAGL inhibitors JZL184, MJN110, and pristimerin alleviated and prevented paclitaxel-induced mechanical allodynia [7,8,9].

The first characterized natural compounds that inhibited MAGL activity are the triterpenes, pristimerin and euphol [10]. They inhibit the activity of MAGL in a reversible manner. Pristimerin inhibited MAGL with higher potency than euphol [10]. Other studies have shown that other triterpenes such as α-amyrin and β-amyrin inhibit MAGL activity in vitro but with lower potency or effect than pristimerin [11]. It has also been shown recently that pristimerin inhibits MAGL activity in purified human MAGL in vitro and in the brain and paw skin tissues of mice [8]. Pristimerin also prevented the development of paclitaxel-induced mechanical allodynia [8].

Triterpenes are bioactive secondary metabolites found in various herbal plants used in ethnomedicine such as *Swertia mileensis, Euphorbia kansui, Alismatis rhizome, Panax ginseng* C. A. Meyer [12,13,14,15,16]. They have been found to have many biological effects including anticancer, osteogenic, antidiabetic, analgesic, and anti-inflammatory activities, amongst many others [12,13,14,15,16]. Various studies have also shown that triterpenes have antinociceptive activities and can alleviate pain symptoms associated with inflammation and neuropathic pain [8,11,17,18]. Some of the antinociceptive and antiallodynic activities of these triterpenes, such as β-amyrin and pristimerin, have been attributed to their inhibitory effects on MAGL activity, leading to an increase in the levels of 2-AG [8,11].

There is a possibility that other triterpenes found in plants used in ethnomedicine inhibit MAGL activity. To reduce the cost and time of evaluating triterpene activity in in vitro and in vivo experiments, it would be necessary to evaluate them in silico to enable selection of triterpenes with the best MAGL inhibitory activities. Molecular docking has been used to evaluate the interaction of triterpenes, as well as other molecules, with MAGL [10,19,20,21]. Because of the presence of freely available software and servers, molecular docking is a cheap and easy way to evaluate the affinity of ligands to their protein targets [22,23,24]. Molecular dynamics simulations (MDSs) provide computational validation of molecular docking studies. However, no studies have been carried out that correlate the affinity of triterpenes to MAGL obtained by molecular docking and MDS to the inhibitory effects of triterpenes on MAGL activity in vitro. If there is a correlation between affinity and free energy binding scores of triterpenes to MAGL and their potency of inhibiting MAGL in vitro, it would make it easy to screen the triterpenes.

In this study, the free energy of binding of triterpenes on MAGL was computed. The affinity (kcal/mol) was calculated using SwissDock server, and docking was carried out by using a publicly available docking server, CB-Dock2, from the Young Cao Lab, [23] and also by PyRx-0.8. These were compared with their in vitro inhibition of MAGL activity. MDSs were carried out to validate the molecular docking results. The triterpenes that were evaluated were chosen based on the presence of the IC_50_ values of MAGL inhibition in previous studies in comparison to pristimerin [10,11].

## 2. Materials and Methods

### 2.1. Literature Search

A search of articles in the U.S. National Library of Medicine, Washington, DC (MED-LINE-PubMed) was conducted for this study up to 21 June 2024. The following keywords were proposed and searched for in PubMed: “pristimerin”, “triterpene”, “euphol’, “monoacylglycerol lipase”, “inhibitor”, and “inhibition”. Six searches were carried out using different combinations of the keywords or phrases (“pristimerin” OR “euphol” OR “triterpene”) AND (“monoacylglycerol lipase”) AND (“inhibitor” OR “inhibition”).

Only primary research articles that reported the evaluation of the inhibition of MAGL activity, and the IC_50_, by triterpenes (including pristimerin and/or euphol) were included. Review articles were excluded as well as articles that did not evaluate MAGL inhibition by pristimerin and/or euphol.

### 2.2. Molecular Docking and Molecular Dynamics Simulations

The three-dimensional (3D) structures of the triterpenes α-amyrin (PubChem CID 73170), β-amyrin (PubChem CID 73145), euphol (PubChem CID 441678), and pristimerin (Pub-Chem CID 159516) were downloaded from PubChem database in .sdf 3D conformer format (https://pubchem.ncbi.nlm.nih.gov/, accessed on 15 June 2024). The X-ray crystal structure of human MAGL in complex with an inhibitor, compound 3l (PDB id: 5ZUN), was retrieved from Protein Data Bank (PDB, https://www.rcsb.org/, accessed on 19 June 2024), in .pdb format.

Similar to our recent study [25], CB-Dock2 (https://cadd.labshare.cn/cb-dock2/index.php, accessed on 9 August 2024), a publicly available docking server from the Yang Cao Lab was used for molecular docking using the auto blind docking option, as described previously [23]. The docked protein–ligand complexes were downloaded. The lowest Vina scores (highest binding affinity), based on the latest AutoDock Vina, as well as the contact residues in the MAGL cavity for each compound were obtained.

The studied compounds and the protein were prepared for docking. The free energy of binding was computed, and the affinity (kcal/mol) was calculated using SwissDock server [26,27]. Docking was carried out by PyRx-0.8 [28]. SwissDock and PyRx-0.8 also utilized AutoDock tools and AutoDock Vina. CB-Dock2 and PyRx-0.8 were chosen for docking because they both utilize AutoDock Vina for validation of the docking process, and also because of our experience from previous studies [25,29].

The affinity, free energy of binding, and docking scores by both PyRx-0.8 and CB-Dock2 (AutoDock vina scores) of the triterpenes were used for comparison of the triterpenes.

Preliminary MDSs were conducted for the best docking pose for the four ligand–MAGL complexes to enable RMSF calculation. The best conformation from each docking process was kept inside the active site. All hydrogens were added, and energy minimization was computed. The solvent molecules that were in the system were deleted before solvation and salt atoms were added to ensure complete neutralization of the system. Solvent atoms were added to surround the system in a spherical shape. The protein–ligand complex was surrounded by a sphere shape of solvent (water) and NaCl was used as a salt to neutralize the charged system. Amber12: EHT was selected as an all-atom forcefield. All bonds, van der Waals, electrostatics, and restraints were enabled. Energy minimization was performed. Root mean square deviation gradient was 1.0. Start time was zero. The MDS protocol was carried out using NPA (Nose–Poincare–Andersen) as a method for solving the equation of motion. Temperature was 300 K and time scale was 50 ns.

### 2.3. Correlation of Affinity, Free Energy of Binding, and Docking Scores and IC_50_ of MAGL Inhibition Data from In Vitro Studies

The GraphPad Prism software (version 10.3) was used to assess the association between the affinity, free energy of binding, and docking scores by both PyRx-0.8 and CB-Dock2 (AutoDock vina scores) of the triterpenes and their in vitro IC_50_ of MAGL inhibition data, obtained from studies by King and colleagues and Chicca and colleagues [10,11], using simple linear regression analyses.

### 2.4. In Silico Prediction of the Pharmacokinetic Parameters

All prediction was carried out using pkCSM (https://biosig.lab.uq.edu.au/pkcsm/). The databases of experimentally measured ADMET properties are widely available. pkCSM applied a graph-based structural signature to predict ADMET properties of novel compounds when compared to the reported compounds in the databases.

## 3. Results

### 3.1. Articles That Reported the Evaluation of the Inhibition of MAGL Activity by Triterpenes, Including Euphol and/or Pristimerin

**Figure A1:**
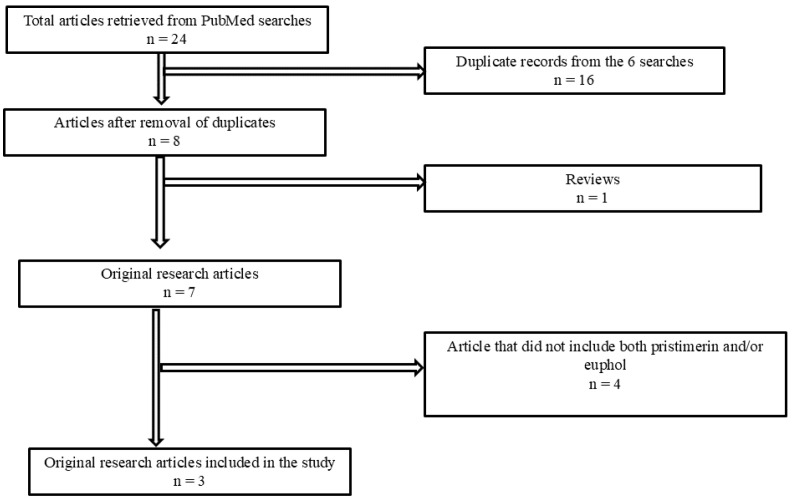
Flow diagram of the different phases of the systematic article selection.

The PubMed searches produced twenty-four articles, which were reduced to eight articles after the removal of duplicates. Five articles were excluded for several reasons, detailed in Table A1 and Figure A1, which shows the study flow information. Thus, three articles from PubMed searches fit the inclusion criteria. King and colleagues studied the inhibition of MAGL activity by pristimerin and euphol on purified recombinant rat MAGL and non-purified (cell lysates of MAGL-transfected HeLa cells) and found that pristimerin was more potent than euphol in inhibiting MAGL activity; see Table 1 [10]. Chicca and colleagues studied the inhibition of MAGL activity by α-amyrin, β-amyrin and pristimerin on purified recombinant human MAGL and found that pristimerin was more potent than β-amyrin, and the latter was more potent than α-amyrin (Table 1; [11]). We, Al-Romaiyan and Masocha, studied the inhibition of MAGL activity by betulinic acid, cucurbitacin B, euphol and pristimerin on recombinant human MAGL, non-purified mouse brain and non-purified mouse paw skin [8]. Only pristimerin had a concentration-dependent inhibition of recombinant human MAGL, as well as on non-purified mouse brain and non-purified mouse paw skin activity. Similar to the study by King and colleagues [10], Al-Romaiyan and Masocha found that pristimerin had more effect against purified recombinant MAGL than non-purified MAGL [8]. At a fixed concentration of 1 µM, pristimerin (50.3150 ± 4.0452%) inhibited recombinant human MAGL more than euphol (15.932 ± 8.654%) [8], which is in line with the results from previous studies [10].

### 3.2. Molecular Docking and Molecular Dynamic Simulations of Triterpenes on MAGL

**Figure 1:**
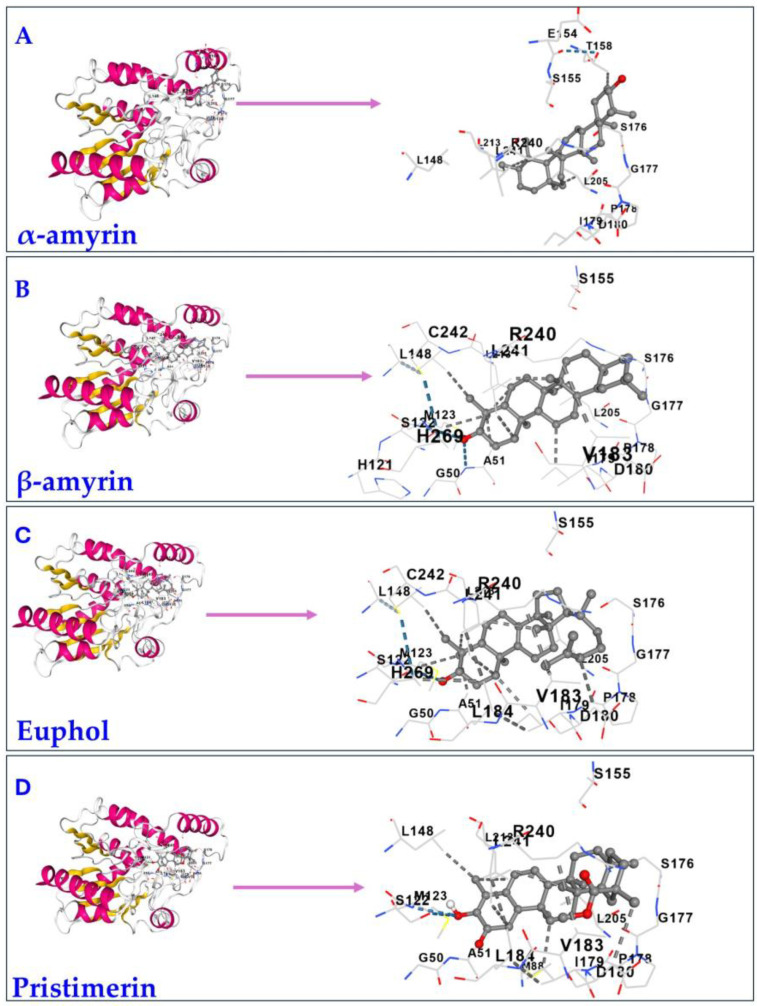
Illustration of the molecular docking of triterpenes to MAGL (5ZUN). α-amyrin, β-amyrin, euphol, and pristimerin interact with the same MAGL cavities. The best potential cavity regions on MAGL were detected by a structure-based approach. The interactive visualization for the 3D structure of MAGL with (**A**) α-amyrin, (**B**) β-amyrin, (**C**) euphol, and (**D**) pristimerin bound to the cavity. The receptor, MAGL, was coloured by secondary structure. The docking files have been uploaded to Zenodo. https://doi.org/10.5281/zenodo.16890027.

The results of the molecular docking of the four triterpenes, α-amyrin, β-amyrin, euphol, and pristimerin on MAGL using CB-Dock2 are shown in Table 2 and Figure 1. All four triterpenes docked to the same cavity of MAGL and similar contact residues, with high affinity. The affinity of the triterpenes based on Vina scores was pristimerin (−11.5 kcal/mol) > euphol (−10.7 kcal/mol) > β-amyrin (−8.8 kcal/mol) ≥ α-amyrin (−8.6 kcal/mol). Thus, pristimerin had the highest binding affinity, better than euphol, α-amyrin and β-amyrin. The ligands bound to nearly the same amino acid contact residues (high-lighted in red). α-amyrin, β-amyrin and euphol bound to four other amino acids (high-lighted in turquoise), to which pristimerin did not bind.

**Figure 2:**
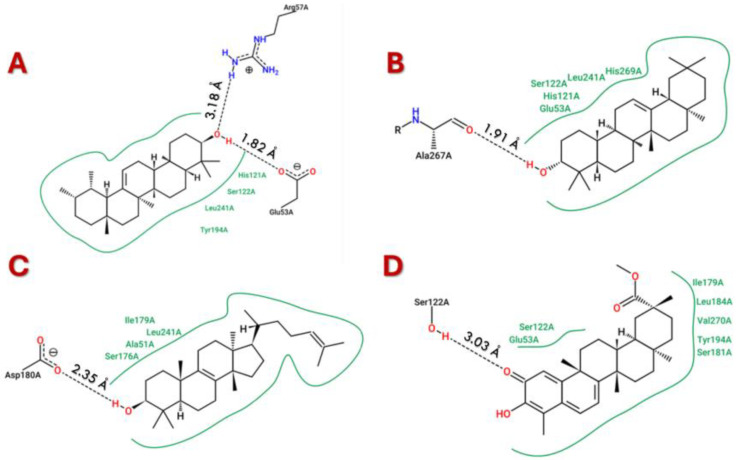
The best docking pose for (**A**) α-Amyrin–MAGL complex, (**B**) β-Amyrin–MAGL complex, (**C**) Euphol–MAGL complex, and (**D**) Pristimerin–MAGL complex.

According to the docking scores (Table 3), pristimerin had the top ranked affinity (52.75 kcal/mol), free energy of binding (−9.32 kcal/mol), and docking score by both PyRx-0.8 (−10.83 kcal/mol) and CB-Dock2 (−11.5 kcal/mol), which is an excellent computational validation of the results. The main interactions of α-amyrin were the hydrogen bond between -NH2 group of Arg57 and the oxygen atom of -OH group, in addition to another hydrogen bond between Glu53 carboxylic group -C=O and the hydrogen atom of -OH group (Figure 2A). On the other hand, the hydroxyl group of β-amyrin interacted by hydrogen bond formation with Ala267 with possible hydrophobic interactions with Leu241 (Figure 2B). The -OH group plays an important role in euphol’s interactions through the formation of ahydrogen bond with Asp180 and a very good placement and fitting for the alkenyl side chain (Figure 2C). Pristimerin showed a hydrogen bond between -C=O and Ser122 with possible hydrophobic interactions with Leu184, Ile179, Val270, and Tyr194 (Figure 2D).

**Figure 3:**
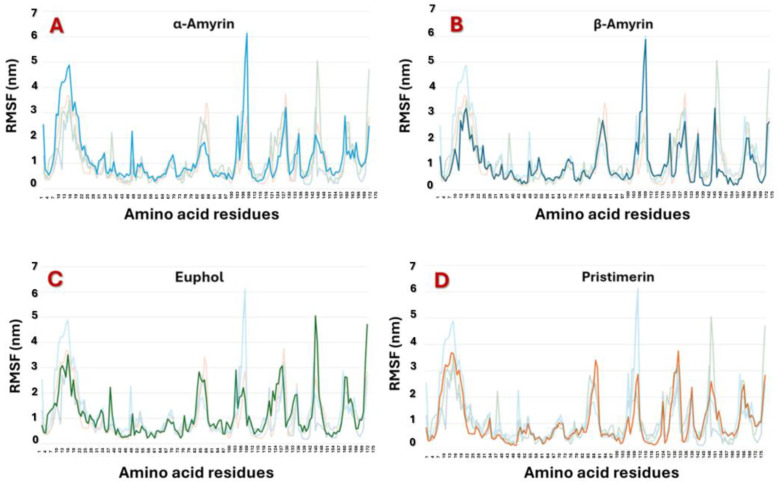
Root mean square fluctuation (RMSF) of (**A**) α-Amyrin–MAGL complex, (**B**) β-Amyrin–MAGL complex, (**C**) Euphol–MAGL complex, and (**D**) Pristimerin–MAGL complex.

**Figure 4:**
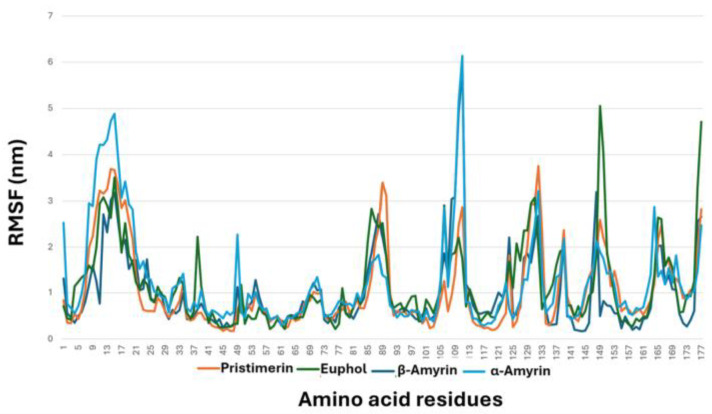
Collective root mean square fluctuation (RMSF) graph of the studied MAGL–ligand complexes.

The compounds studied were subjected to an MDS study over 50 ns. During this period, pristimerin showed the highest stability and reached the steady state after 20 ns with the lowest root mean square fluctuation (RMSF), especially at the binding site (docking site) (Figure 3D), when compared to the other three compounds: α-amyrin, β-amyrin, and euphol (Figure 4).

### 3.3. Comparison of Affinity, Free Energy of Binding and Docking Scores by Both PyRx-0.8, and CB-Dock2 and IC_50_ of MAGL Inhibition Data from In Vitro Studies

**Figure 5:**
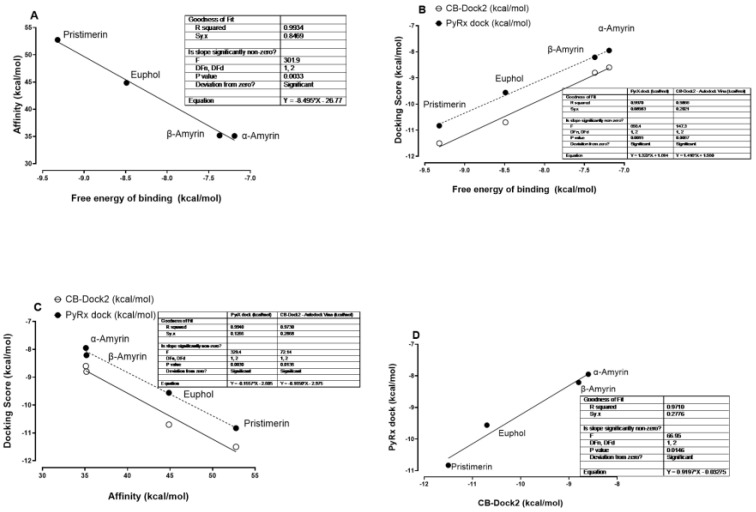
Relationships of α-amyrin, β-amyrin, euphol and pristimerin scores on interaction with MAGL for affinity, free energy of binding, and docking score by both PyRx-0.8 and CB-Dock2. Correlation of (**A**) affinity and free energy of binding, (**B**) docking scores and free energy of binding, (**C**) affinity and docking scores, and (**D**) PyRx-0.8 dock and CB-Dock2 docking scores. Simple linear regression analyses performed using GraphPad Prism software (version 10.3). The x- and y-axes represent predicted values.

Free energy of binding had a significantly negative correlation with affinity and positive correlation with docking scores (Figure 5A,B). Affinity had a significantly negative correlation with docking scores (Figure 5C). The PyRx-0.8 and CB-Dock2 docking scores had a significantly positive correlation (Figure 5D). This was an excellent computational validation of the results of the different methods and software.

**Figure 6:**
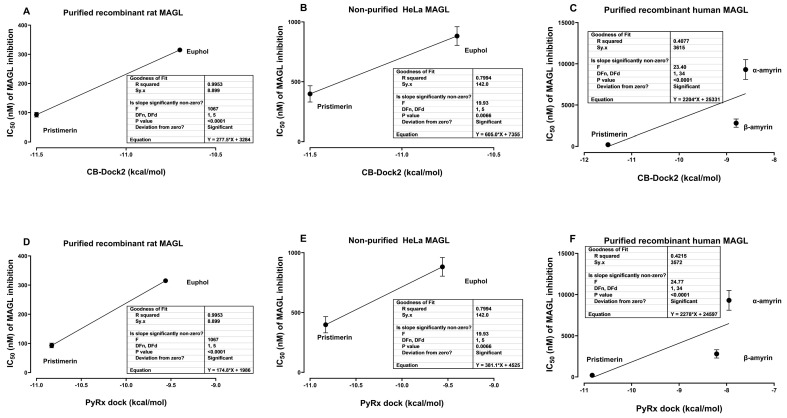
Correlation of CB-Dock2 and PyrX dock docking scores with IC_50_ of MAGL inhibition. Correlation of Vina scores of euphol and pristimerin (obtained from molecular docking using CB-Dock2) with the IC_50_ of MAGL inhibition on (**A**) purified recombinant rat MAGL and (**B**) non-purified HeLa MAGL using data from King and colleagues [10]. (**C**) Correlation of Vina scores of α-amyrin, β-amyrin and pristimerin with the IC_50_ of MAGL inhibition on purified recombinant human MAGL using data from Chicca and colleagues [11]. Correlation of PyrX dock scores of euphol and pristimerin with the IC_50_ of MAGL inhibition on (**D**) purified recombinant rat MAGL and (**E**) non-purified HeLa MAGL using data from King and colleagues [10]. (**F**) Correlation of PyrX dock of α-amyrin, β-amyrin and pristimerin with the IC_50_ of MAGL inhibition on purified recombinant human.

Molecular docking scores with CB-Dock2 and PyRx-0.8 showed that pristimerin had higher binding affinity (docking scores of −11.5 and −10.83 kcal/mol, respectively) than euphol (docking scores of −10.7 and −9.56 kcal/mol). The binding affinity of the two triterpenes significantly correlated with the IC_50_ of MAGL inhibition data (*p* < 0.0001) from in vitro studies [10]: 93 ± 8 nM for pristimerin and 315 ± 1 nM for euphol on purified recombinant rat MAGL (Figure 6A,D), and 398 ± 68 nM for pristimerin and 882 ± 78 nM for euphol on non-purified HeLa cell MAGL (Figure 6B,E).

Pristimerin had higher binding affinity (Vina score of −11.5 kcal/mol) than β-amyrin (Vina score of −10.7 kcal/mol), and β-amyrin had higher binding affinity than α-amyrin. The binding affinity of the three triterpenes significantly correlated with the IC_50_ of MAGL inhibition data (*p* < 0.0001) from in vitro studies [11]: 204 ± 16.2 nM for pristimerin, 2800 ± 500 nM for β-amyrin, and 9300 ± 1200 nM for α-amyrin on purified recombinant human MAGL (Figure 6C,F).

### 3.4. Results of In Silico Prediction of Pharmacokinetic Parameters

Pharmacokinetic predictions were carried out using pkCSM (https://biosig.lab.uq.edu.au/pkcsm/) (accessed on 1 August 2025) and the results are included in Table A2.

According to the predicted absorption, distribution, metabolism, excretion, and toxicity (ADMET), all compounds showed almost similar absorption properties (water solubility, Caco2 permeability, intestinal absorption, skin permeability, P-glycoprotein substrate, P-glycoprotein I inhibition, and P-glycoprotein II inhibition properties. All compounds showed high predicted intestinal permeability, as they have Caco2 permeability values > 0.9. The compounds illustrated high skin permeability log Kp values. Regarding the distribution parameters, pristimerin was the only compound to show low steady state volume of distribution (VDss), which means low distribution in tissues rather than plasma. The other three compounds showed high VDss in tissues, which can be affected in the case of patients with renal failure. For the blood–brain barrier (BBB) permeability, alpha- and beta- amyrin, and euphol showed Log BB > 0.3, which means they have good distribution in the brain. Pristimerin had a poor BBB permeability, which means it is poorly distributed in the brain when compared to the other three compounds. All compounds have the same predicted metabolism profile. They all have low maximum tolerated dose (<0.0477 log mg/kg/day). Neither hepatotoxicity nor skin sensitization was detected in the in silico predictions.

## 4. Discussion

MAGL is a target for managing neuropathic pain. A systematic search identified four triterpenes, α-amyrin, β-amyrin, euphol and pristimerin, that had IC_50_ values for inhibition of MAGL activity in vitro. These four triterpenes are found in herbal plants used in ethnomedicine [12,15,30,31]. Molecular docking showed that these four triterpenes bound to the same MAGL cavity and similar amino acid residues with high affinity in the following order using docking scores (binding energy): pristimerin > euphol > β-amyrin > α-amyrin. Simple linear regression analyses showed that there was a significant correlation between the docking scores and the IC_50_ values for inhibition of MAGL activity, i.e., the lower the binding energy (higher affinity), the lower the IC_50_ (more potency). The relationship of the triterpene molecules was consistent in terms of affinity, free energy of binding, and docking scores, which is an excellent computational validation of the results. MDS results showed that pristimerin had the highest stability and reached the steady state after 20 ns with lowest RMSF, especially at the binding site (docking site), when compared to the other three compounds.

The endocannabinoid 2-AG is produced on demand and rapidly metabolized by the enzyme MAGL [4,5,6]. 2-AG has analgesic effects and alleviates neuropathic pain-related allodynia [7,32,33]. In some models of neuropathic pain, there is a deficiency of 2-AG and increased MAGL activity [7,8,34]. Thus, inhibitors of the activity of MAGL, by increasing the levels of 2-AG, have analgesic effects, and prevent or alleviate allodynia [7,8,34]. Several MAGL inhibitors have been synthesized and most inhibit MAGL irreversibly [35]. The triterpenes, such as pristimerin and euphol, inhibit MAGL reversibly and have been shown to prevent and alleviate allodynia [8,10]. Other triterpenes, such as α-amyrin and β-amyrin, also have MAGL inhibitory activity but with less potency than pristimerin [11]. Thus, it is possible that amongst the hundreds of triterpenes in herbal plants, there may be those with MAGL inhibitory activities. It is expensive to evaluate MAGL inhibitory effects of triterpenes in vitro; therefore, in silico screening methods such as molecular docking could be useful to screen large libraries of triterpenes for potentially active and potent congeners.

King and colleagues evaluated the MAGL inhibitory activities of pristimerin and euphol using purified recombinant rat MAGL and non-purified HeLa cell MAGL. Pristimerin and euphol were more potent in inhibiting purified recombinant rat MAGL than non-purified HeLa cell MAGL [10]. Pristimerin was more potent than euphol on inhibiting both purified recombinant rat MAGL and non-purified HeLa cell MAGL [10]. Molecular docking showed that pristimerin had lower binding energy (−11.5 and −10.83 kcal/mol), thus higher binding affinity, than euphol (−10.7 and −9.56 kcal/mol). Simple linear regression analyses showed that there was a significant correlation between the docking scores and the IC_50_ values for inhibition of MAGL activity by pristimerin and euphol. Thus, looking at the molecular docking scores, one could predict which molecule was more potent than the other between pristimerin and euphol. Al-Romaiyan and Masocha also showed that at a concentration of 1 µm pristimerin inhibited human MAGL activity more than euphol [8], in line with the IC_50_ value relationships reported by King and colleagues [10].

Chicca and colleagues evaluated the MAGL inhibitory activities of pristimerin, α-amyrin and β-amyrin using purified recombinant human MAGL. Pristimerin was more potent than β-amyrin, which was more potent than α-amyrin in inhibiting purified recombinant human MAGL [11]. Molecular docking showed that pristimerin had lower binding energy (−11.5 and −10.83 kcal/mol), thus higher binding affinity, than β-amyrin (−8.8 and −8.21 kcal/mol), which had lower binding energy than α-amyrin (−8.6 and −7.95 kcal/mol). Simple linear regression analyses showed that there was a significant correlation between the docking scores and the IC_50_ values for inhibition of MAGL activity by pristimerin, α-amyrin and β-amyrin. Thus, using molecular docking scores, one could predict which molecule would be more potent than the other amongst pristimerin, α-amyrin and β-amyrin. In addition, pristimerin, the most active compound among the tested triterpenes, exhibited a high degree of unsaturation in the A and B rings, resulting in a planar structure for this moiety, a characteristic that the other triterpenes do not share, and this could be another reason for its better activity. This characteristic needs to be further investigated.

The molecular docking studies also show that the trieterpenes interacted with the active site of MAGL. All triterpenes interacted with Ser122 of the catalytic triad, while all others except pristimerin interacted with HIS269, but none interacted with ASP239 [36,37]. On the oxyanion hole, which has influence on catalytic activity, all the triterpenes interacted with Gly50, Ala51, and Met123, but not Gly124 [37,38]. All the triterpenes interacted with PHE159 and ILE179, which are residues in the lid domain, and ALA51, ILE179, LEU213, and LEU241,which have been shown to form hydrophobic interactions with phenyl rings of inhibitors [37,39].

One of the limitations of this study is that only a few data points were available (IC_50_ values of only four triterpenes) and used in each regression, and this could affect the statistical significance and predictive value of the in silico studies. However, using different computational tools and producing similar results suggests that besides limited data availability, the in silico results correlate well with the in vitro data.

## 5. Conclusions

The findings of this study show that molecular docking, using the CB-Dock2 server and PyRx-0.8, correlate with the potency of triterpenes found in herbal plants used in ethnomedicine and their inhibition of MAGL activity in vitro. In terms of affinity and docking scores to MAGL, pristimerin was better than euphol, which was better than β-amyrin, which was better than or equal to α-amyrin, respectively. The IC_50_ values of recombinant human and rat MAGL inhibition found from two publications were in the following order: α-amyrin > β-amyrin > euphol > pristimerin. Linear regression analysis showed that the affinity and docking scores significantly correlated with the IC_50_ of MAGL inhibition. In MDS, pristimerin showed the highest stability and reached the steady state after 20 ns with the lowest RMSF at the binding site, compared to the other triterpenes. Amongst the triterpenes studied, pristimerin was the most potent inhibitor of MAGL and also had the highest affinity in the in silico studies. Thus, molecular docking could be used as a tool for virtual screening of triterpenes as MAGL inhibitors in comparison to pristimerin before wet laboratory experiments to save experimental time and costs of wet laboratory experiments. Importantly, the affinity, free energy of binding, and docking scores by both PyRx-0.8 and CB-Dock2 all showed significant correlation, which is an excellent computational validation of the results.

## Figures and Tables

**Table 1:** IC_50_ values of α-amyrin, β-amyrin, euphol and pristimerin inhibition of MAGL activity.

Triterpene	IC_50_ *	Source/Type of MAGL	Reference
α-Amyrin	9300 ± 1200 nM	Recombinant human MAGL	[11]
β-Amyrin	2800 ± 500 nM	Recombinant human MAGL	[11]
Euphol	315 ± 1 nM	Purified recombinant rat MAGL	[10]
	882 ± 78 nM	Non-purified (cell lysates of MAGL-transfected HeLa cells)	[10]
Pristimerin	93 ± 8 nM	Purified recombinant rat MAGL	[10]
	398 ± 68 nM	Non-purified (cell lysates of MGL-transfected HeLa cells	[10]
	204 ± 16.2 nM	Recombinant human MAGL	[11]
	130 nM	Recombinant human MAGL	[8]
	1600 nM	Non-purified mouse brain	[8]
	596 nM	Non-purified Mouse paw skin	[8]

* IC_50_ values from different studies were obtained under different assay conditions.

**Table 2:** Comparison of the best CB-Dock2 molecular docking Vina scores (kcal/mol) of α-amyrin, β-amyrin, euphol, and pristimerin on MAGL (5ZUN). Cavity volume (1904, Å3), center (x, y, z; 10, 17, −6), and docking size (x, y, z; 23, 23, 23).

Triterpene	Vina Score (kcal/mol)	Contact Residues
α-Amyrin	−8.6	GLY50 ALA51 MET88 SER122 MET123 LEU148 ALA151 ASN152 GLU154 SER155 ALA156 THR158 PHE159 SER176 GLY177 PRO178 ILE179 ASP180 VAL183 LEU184 LEU205 PHE209 GLY210 LEU213 LEU214 ARG240 LEU241 HIS269
β-Amyrin	−8.8	GLY50 ALA51 GLY52 HIS121 SER122 MET123 LEU148 ALA151 ASN152 GLU154 SER155 ALA156 THR158 PHE159 SER176 GLY177 PRO178 ILE179 ASP180 SER181 VAL183 LEU184 LEU205 PHE209 GLY210 LEU213 LEU214 VAL217 ARG240 LEU241 CYS242 HIS269
Euphol	−10.7	GLY50 ALA51 MET88 SER122 MET123 LEU148 LEU150 ALA151 ASN152 GLU154 SER155 ALA156 THR158 PHE159 LYS160 SER176 GLY177 PRO178 ILE179 ASP180 VAL183 LEU184 LEU205 PHE209 GLY210 LEU213 LEU214 VAL217 ARG240 LEU241 CYS242 HIS269
Pristimerin	−11.5	GLY50 ALA51 MET88 SER122 MET123 LEU148 ALA151 SER155 ALA156 THR158 PHE159 SER176 GLY177 PRO178 ILE179 ASP180 VAL183 LEU184 LEU205 GLY210 LEU213 LEU214 VAL217 ARG240 LEU241

**Table 3:** Affinity and docking scores obtained using SwissDock, PyRx-0.8 dock, and CB-Dock2. All three virtual screening software/servers utilize AutoDock Vina docking system.

Scores	α-Amyrin	β-Amyrin	Euphol	Pristimerin
PubChem CID	73,170	73,145	441,678	159,516
Free energy of binding (kcal/mol)	−7.19	−7.37	−8.49	−9.32
Affinity (kcal/mol)	35.10	35.17	44.86	52.75
PyRx-0.8 dock (kcal/mol)	−7.95	−8.21	−9.56	−10.83
CB-Dock2 (kcal/mol)	−8.6	−8.8	−10.7	−11.5

## Data Availability

Data will be made available on request. The docking files have been uploaded to Zenodo. https://doi.org/10.5281/zenodo.16890027.

## Appendix B

**Table A1:** Articles found in PubMed that were excluded from the analysis.

Article Number	Primary Reason for Exclusion	Article Type	Reference
1.	Review article	Review	[25]
2.	Did not evaluate MAGL inhibition by either pristimerin or euphol	Original research	[40]
3.	Did not evaluate MAGL inhibition by either pristimerin or euphol	Original research	[41]
4.	Did not evaluate MAGL inhibition by either pristimerin or euphol	Original research	[42]
5.	Did not evaluate MAGL inhibition by either pristimerin or euphol	Original research	[43]

## Appendix C

**Table A2:** In silico prediction of the pharmacokinetic parameters.

	Model Name	α-Amyrin	β-Amyrin	Euphol	Pristimerin
**Absorption**	Water solubility (log mol/L)	−6.804	−6.84	−7.53	−5.902
	Caco2 permeability (log Papp in 10–6 cm/s)	1.288	1.287	1.218	1.077
	Intestinal absorption (human) (% Absorbed)	97.574	97.246	93.056	100
	Skin Permeability (log Kp)	−2.898	−2.894	−2.933	−2.649
	P-glycoprotein substrate (Yes/No)	No	No	No	No
	P-glycoprotein I inhibitor (Yes/No)	Yes	Yes	Yes	Yes
	P-glycoprotein II inhibitor (Yes/No)	Yes	Yes	Yes	Yes
**Distribution**	VDss (human) (log L/kg)	0.443	0.446	0.692	−0.353
	Fraction unbound (human)	0	0	0	0
	BBB permeability (log BB)	0.735	0.728	0.702	−0.344
	CNS permeability (log PS)	−1.971	−1.971	−2.247	−1.102
**Metabolism**	CYP2D6 substrate (Yes/No)	No	No	No	No
	CYP3A4 substrate (Yes/No)	Yes	Yes	Yes	Yes
	CYP1A2 inhibitor (Yes/No)	No	No	No	No
	CYP2C19 inhibitor (Yes/No)	No	No	No	No
	CYP2C9 inhibitor (Yes/No)	No	No	No	No
	CYP2D6 inhibitor (Yes/No)	No	No	No	No
	CYP3A4 inhibitor (Yes/No)	No	No	No	No
**Execretion**	Total Clearance (log mL/min/kg)	0.119	−0.044	0.403	−0.034
	Renal OCT2 substrate (Yes/No)	No	No	No	No
**Toxicity**	Max. tolerated dose (human) (log mg/kg/day)	0.203	0.212	−0.35	0.324
	Oral Rat Acute Toxicity (LD50) (mol/kg)	2.156	2.169	1.867	3.234
	Oral Rat Chronic Toxicity (LOAEL) (log mg/kg_bw/day)	0.908	0.926	0.775	1.757
	Hepatotoxicity (Yes/No)	No	No	No	No
	Skin Sensitisation (Yes/No)	No	No	No	No
